# Comprehensive Histological and Immunochemical Forensic Studies in Deaths Occurring in Custody

**DOI:** 10.1155/2017/9793528

**Published:** 2017-03-12

**Authors:** Kenneth Nugent, Menfil A. Orellana-Barrios, Dolores Buscemi

**Affiliations:** ^1^Department of Internal Medicine, Texas Tech University Health Sciences Center, Lubbock, TX, USA; ^2^Division of Cardiology, UFJAX, Jacksonville, FL, USA

## Abstract

In-custody deaths have several causes, and these include homicide, suicide, natural death from chronic diseases, and unexplained death possibly related to acute stress, asphyxia, excited delirium, and drug intoxication. In some instances, these deaths are attributed to undefined accidents and natural causes even though there is no obvious natural cause apparent after investigation. Understanding these deaths requires a comprehensive investigation, including documentation of circumstances surrounding the death, review of past medical history, drug and toxicology screens, and a forensic autopsy. These autopsies may not always clearly explain the death and reveal only nonspecific terminal events, such as pulmonary edema or cerebral edema. There are useful histologic and biochemical signatures which identify asphyxia, stress cardiomyopathy, and excited delirium. Identifying these causes of death requires semiquantitative morphologic and biochemical studies. We have reviewed recent Bureau of Justice Statistics on in-custody death, case series, and morphological and biochemical studies relevant to asphyxia, stress cardiomyopathy, and excited delirium and have summarized this information. We suggest that regional centers should manage the investigation of these deaths to provide more comprehensive studies and to enhance the expertise of forensic pathologists who would routinely manage potentially complex and difficult cases.

## 1. Introduction

In-custody deaths represent an important problem to the judicial system and to the decedent's family members [1, 2]. These cases often occur without an obvious cause for the deaths and are labeled death by* natural causes*. Potential explanations include trauma, suicide, excited delirium, mechanical asphyxia, stress cardiomyopathy, and seizures. In younger decedents, underlying medical disorders are unlikely to contribute to death, in contrast to older individuals with chronic medical conditions. The evaluation of these cases requires a comprehensive approach, but there is a frequent concern that the forensic pathology is casual and biased towards the judicial system [3]. It is particularly difficult to establish the cause of death when the decedent has excited delirium, stress cardiomyopathy, or sudden death from epilepsy. In addition, it may be difficult to establish the cause of death when the etiology involves mechanical asphyxia done in a manner which creates little postmortem evidence of external trauma. Forensic pathologists need to do a thorough external and internal organ evaluation and selected histologic studies. It is possible that molecular studies can help identify unusual causes of death which might be attributed to natural causes. In this review, we consider histological studies and molecular studies which could contribute to the identification of death associated with excited delirium, stress cardiomyopathy, drowning, asphyxia, and possibly seizures.

## 2. Methods

PubMed was searched using the MeSH terms forensic pathology, death, sudden, asphyxia, drowning, pulmonary surfactant-associated proteins, heat shock protein, and stress cardiomyopathy and the text terms in-custody, excited delirium, contraction band necrosis, and epilepsy. The main interest was to identify literature relevant to sudden death and not death associated with acute hospital care. This information was summarized into a narrative review.

## 3. Results

### 3.1. National Statistics

Between 2003 in 2009, 4,813 arrest-related deaths were reported to the Bureau of Justice Statistics. Approximately 60% of these deaths were classified as homicides by law enforcement personnel, and 40% were attributed to suicide (11%), intoxication (11%), accidental injury (6%), and natural causes (5%) [10].

### 3.2. Case Series

Five recent case series have reported in-custody or in-jail deaths (Table 1) [4–8]. These studies include 1898 decedents. There were a disproportionate number of deaths secondary to suicide in one case series [8]. The totals from these series including the one with a large number of suicides indicate that approximately 46% of the decedents died from natural causes and 46% died from suicide. This is a very high percentage of suicides and warrants more study. These suicides most likely reflect, in part, aberrant responses to extreme circumstances related either to the facility and prisoner management policies or to underlying psychiatric or drug-related disorders [9, 11]. A small percentage (3.6%) died from accidents in prison or jail. However, very few details are provided about these accidents.

### 3.3. Pulmonary Pathology

Several circumstances can cause sudden death secondary to respiratory failure; mechanical asphyxia is a potential concern in all in-custody deaths. In many cases, external and internal signs of trauma provide the correct diagnosis. However, additional histological studies and molecular studies can help identify death secondary to asphyxia and drowning.

Klysner and coworkers studied 33 cases of homicidal strangulation in Denmark [12]. Information from the autopsies included gross examination of the lungs and computerized image analysis using stereological methods of routine hematoxylin and eosin stained histologic sections. The lungs from decedents with homicidal strangulation had increased alveolar size, approximately twice that of control subjects who died naturally from cerebral causes. Other histologic findings, such as edema and hyperemia, did not distinguish the lungs in cases with death from strangulation from the lungs in cases with death from natural causes. Giorgetti et al. studied 14 cases of death associated with asphyxia secondary to hanging or drowning and 13 cases of sudden natural death [13]. Tissue sections were analyzed using computerized image analysis to determine the percentage of pulmonary parenchyma in any given a lung section. Subjects who died from natural causes and drowning had relatively high levels of internal homogeneity compared to subjects who had been hanged. The range of fractional areas with lung parenchyma was much broader in hanged subjects, and the standard deviation was much higher. This study suggested that it is possible to distinguish between subjects who drowned and subjects who died from a natural cardiac death by computerized analysis of the lung parenchyma. However, it is not possible to distinguish between subjects who were hanged and subjects who died from a natural death because of the significant variability of the findings in the subjects who were hanged. Delmonte analyzed 167 lungs from decedents who died from aspiration, suffocation, drowning, and strangulation using semiquantitative analysis of lung sections [14]. Victims of suffocation had ductal overinflation and interstitial edema. Strangulation victims had areas of bronchiolar constriction and dilation associated with areas of alveolar collapse and overinflation and alveolar hemorrhage. They concluded that semiquantitative analysis of the lung provides supplemental information to identify the cause of asphyxia.

Zhu and colleagues have reported several studies evaluating the utility of surfactant protein A staining in victims with acute respiratory death. They found significant increases in surfactant protein A staining in subjects with mechanical asphyxia from strangling, throttling, smothering, and choking [15]. Over 60% of the autopsy specimens revealed massive aggregates of surfactant protein A. Surfactant protein A aggregates were also found in victims who died in fires and in both freshwater and seawater drowning [16, 17]. Pérez-Cárceles et al. also detected massive aggregates of SP-A in deaths secondary to drowning and other causes of asphyxia, such as hanging, suffocation, and aspiration [18]. These aggregates can develop over very short periods of hypoxic stress (less than 30 minutes). Cecchi et al. studied 34 medical-legal autopsy cases with acute mechanical asphyxia. These cases had intra-alveolar granular deposits of surfactant A and hypoxia-induced factor 1-*α* in small, medium, and large pulmonary vessels [19]. Consequently, the presence of aggregates of surfactant protein A would suggest mechanical asphyxia or drowning, and their absence would suggest an alternative cause of death. Messenger RNA has also been studied in these cases, but there is limited information with this method [20].

### 3.4. Cardiac Pathology

Cardiac deaths in situations of extreme stress may occur secondary to acute myocardial infarction (AMI) in decedents with chronic atherosclerotic coronary disease, stress cardiomyopathy, and cardiac arrhythmias [21]. In subjects with obstructive atherosclerosis, pathological inspection of the myocardium and coronaries can usually clearly identify coronary disease and myocardial infarctions [22, 23]. A notable exception occurs when early myocardial infarction results in sudden cardiac death leaving only evidence of intracoronary thrombi [24]. Stress cardiac cardiomyopathy can be identified by histological and biochemical changes even in the absence of infarction [25]. Myocardial fibrosis, hypertrophy, and interstitial hypercellularity may be substrates for arrhythmias [26] and chronic tachycardia causes changes in ventricular wall thickness [27]. However, there is no histologic signature for acute arrhythmias.

The histological finding of contraction band necrosis (CBN) has been associated with myocardial cell injury and death, particularly related to exposure to high levels of catecholamines (endogenous or exogenous) and transient ischemia with subsequent reperfusion. Contraction band necrosis describes a focal alteration in muscle architecture due to hypercontraction of myofibrils in myocardial cells, producing dense groupings of myofibrils interspersed between clear areas of muscle, barren of myofibrils due to lysis [28]. Contraction band necrosis attributed to high catecholamine levels has been reported in several circumstances of extreme physiological stress, including, stroke [29], trauma [30], meningitis [31], drowning [32], homicide [33], and cocaine abuse [34]. In animals, the condition of “capture cardiomyopathy” has been described in mammals, leading to acute and subacute death and has been proposed as a model of catecholamine induced cardiomyopathy in humans [35, 36]. Although the specific mechanism of CBN is not clearly understood, it may relate to high catecholamine levels inducing cellular calcium dysregulation and free radical production [37]. Contraction band necrosis is also known as myofibrillar degeneration and coagulative myocytolysis [28].

Cebelin and Hirsch reported that victims of homicidal assaults who do not have significant internal injuries often have CBN with stress cardiomyopathy; this occurred in 11 of 15 victims in this study [33]. These contraction bands likely reflect the acute effects of high levels of catecholamines on the myocardium and can develop after 5 to 10 minutes of high dose catecholamine infusions in experimental animals [38]. Victims of trauma, including stab wounds, falls, traffic accidents, and head injuries, often develop contraction band necrosis and have diffusion of calmodulin in the myocardium. These changes were found in 11 victims out of 24 autopsies in subjects who had no underlying myocardial disease and presumably represent severe physiologic stress associated with the events surrounding their deaths [30]. All decedents with contraction bands had diffusion of calmodulin into the myocardium.

Duflou et al. found CBN in both fatal air crash victims and suicidal hanging victims; widespread CBN was more common in air crash victims [39]. Krexi et al. did a retrospective study of 110 cases of sudden cardiac death related to stressful events, such as altercations, physical restraint, job stress, and car accidents [40]. The majority of these decedents were male with a mean age of 36 ± 16 years. Fifty-three percent of these cases had negative autopsies with morphologically normal hearts, suggesting that the deaths were secondary to stress induced cardiac arrhythmias or stress related cardiomyopathy. Cardiac cardiomyopathy and coronary artery disease were found in the minority of cases. Maréchaux et al. found contraction bands in a 40-year-old woman who died acutely from a subarachnoid hemorrhage [41]. She had echocardiographic evidence of inverted takotsubo cardiomyopathy, and histologic studies revealed contraction bands only in the involved myocardial segments. Baroldi et al. studied the histologic forms of contraction bands and measured their density per unit area of myocardial tissue in a large spectrum of human diseases, including 170 accidental deaths [42]. Based on this study, they encouraged the use of the words “contraction band necrosis” for a specific morphofunctional entity caused by increased concentrations to catecholamines, and they calculated a histologic threshold of 37 ± 7 foci per 100 m^2^ tissue as an indication of severe sympathetic stress.

The information in the above paragraphs describes the development of stress cardiomyopathy with contraction band necrosis in unusual circumstances, such as fatal air crashes, hanging, and severe trauma. Clinicians, especially cardiologists, frequently manage patients who present to emergency centers with acute coronary syndromes but actually have stress cardiomyopathy or takotsubo cardiomyopathy [43]. The typical patient with takotsubo cardiomyopathy is a middle-aged woman who presents with chest pain or dyspnea following a severe psychological or physical stress. These patients have characteristic changes on echocardiography which usually reveal hypokinesia or akinesia of the apex of the left ventricle resulting in apical ballooning. The extent of myocardial dysfunction is outside the distribution of a single coronary vessel, and most of these patients have patent coronary arteries on angiography. Abraham and coauthors studied nine cases of stress cardiomyopathy induced by the intravenous administration of epinephrine (six cases) and dobutamine (three cases) for diagnostic or surgical procedures [44]. These patients developed apical (three cases), midventricular (two cases), and basal (4 cases) ballooning. The median initial ejection fraction was 35%; the ejection fraction at follow-up was 55%. The pathophysiology in stress cardiomyopathy involves high levels of circulating catecholamines from the adrenal glands and local catecholamines (especially norepinephrine) released by sympathetic nerves in the myocardium. High levels of catecholamines shift beta receptor function from a Gs stimulatory pathway to a Gi inhibitory pathway to produce a negative inotropic, noncontractile state (i.e., myocardial stunning) [45, 46]. The Gi pathway is associated with myocardial cell protection through the activation of antiapoptotic cellular processes. Consequently, the pathophysiology of stress cardiomyopathy likely reflects differences in local concentrations of catecholamines in the myocardium, the location and density of beta receptors, and receptor sensitivity to catecholamines [46]. There does not appear to be any difference in beta receptor polymorphisms in patients with stress cardiomyopathy when compared to patients with ischemic cardiac events [47, 48]. Reactive oxygen species generated by autooxidation of catecholamines also cause direct damage to myocytes. These patients usually have a good prognosis, and myocardial function returns to normal in several weeks. Consequently, pathologic studies in these patients are limited. They have contraction band necrosis with variable amounts of myonecrosis; some cases also have interstitial inflammatory cells, such as neutrophils and lymphocytes [41, 49, 50]. D'Errico et al. reported histologic studies in two fatal cases of stress cardiomyopathy, and their immunochemical studies showed an “intense and massive expression of beta-1 adrenergic receptors in the deep layers of the myocardium and in the subendocardial layer” [50].

More recently, contraction bands have been further characterized using myocardium immunohistochemistry. Morita examined different forms of CBN by determining the expression and distribution of complement component C9 (CCC9) and Sirtuin 1 (SIRT1) in 30 cardiac tissues taken from 30 autopsy cases [41]. Although their sample size is too small to create a generalizable classification based on CCC9 or SIRT1 reactivity, their study demonstrates a promising technique to differentiate CBN due to myocardial infarction (i.e., CCC9+/SIRT1−) from other etiologies of contraction bands. Additionally, immunohistochemistry may allow the important differentiation of artefactual contraction bands generated during specimen handling. These determinations are important from a forensic science standpoint (particularly when cause of death cannot be determined otherwise) and may avoid misclassification of cardiac death due to AMI based solely on the presence of contraction bands found in standard morphology examinations.

In summary, sudden death associated with severe stress and presumed sympathetic overload can occur in decedents with normal hearts. Histologic evaluation of these hearts should reveal CBN associated with altered Ca^++^ metabolism, diffusion of calmodulin into the myocardium, and free radical production, which indicate the possibility of acute stress cardiomyopathy as the cause of death.

### 3.5. CNS Pathology

In-custody deaths are periodically attributed to excited delirium [51]. Subjects with this clinical syndrome have delirium, agitation, hallucinations, disorientation, violent and bizarre behavior, insensitivity to pain, hyperthermia, and increased strength. This can develop as a consequence of drugs, especially cocaine, psychiatric disorders including schizophrenia, and extreme physical exertion. Subjects with this diagnosis are usually young men often on drugs who have had prolonged physical confrontation with police and required restraints and/or control with electronic weapons. They die with sudden cardiovascular collapse; the cause of death is unknown. However, this conclusion that excited delirium can cause in-custody deaths has been controversial. Families of decedents with in-custody deaths and civil rights organizations have argued that this diagnosis is used to obscure police abuse and inappropriate use of force [52]. Critics have also noted that there is no official diagnosis in the* International Classification of Disease*, Ninth Revision (ICD-9), for excited delirium. The American College of Emergency Physicians created a task force of experts in the field of excited delirium which published a report in 2012 [53]. This panel concluded that excited delirium is a clinical entity and noted that these subjects have typical clinical features but have a complicated differential diagnosis which makes management in emergent situations difficult. This diagnosis is also recognized by the National Association of Medical Examiners and used by medical examiners. Excited delirium is often associated with cocaine toxicity and can be associated with synthetic cannabinoids and bath salts [54–56]. There is no standardized well accepted treatment approach to these patients.

Mash has studied brain tissue from 90 decedents with excited delirium and reported increased levels of heat shock protein 70 messenger RNA and decreased levels of dopamine transporter transcripts [57]. These changes may represent a molecular signature for excited delirium; there are no pathognomonic gross or microscopic autopsy findings. However, Mash has questioned the utility of using elevated messenger RNA levels for heat shock protein as a molecular signature in patients with excited delirium [58]. They reported two patients who had excited delirium but normal levels of messenger RNA for heat shock protein and suggested that elevated levels of heat shock protein reflected the duration of survival following police confrontation or the initiation of medical care. Mash reviewed this disorder in 2016 and described excited delirium as a syndromal disorder of dysregulated dopamine in the central nervous system [59]. She suggested that victims dying with excited delirium have lost dopamine transporter regulation at the synaptic level, resulting in dopamine overflow in central nervous system synapses. This has multiple effects, including agitation, paranoia, violent behavior, and alterations in heart rate, respiration, and core body temperature. This syndrome can develop as a consequence of drug abuse, especially cocaine, withdrawal from psychiatric medicines, alcohol withdrawal, and acute mania with paranoia.

Another potential consideration in patients with unexplained death is unexpected death in epilepsy [60]. Some patients with seizure disorders die suddenly, presumably secondary to uncontrolled seizures. This typically occurs in men with subtherapeutic anticonvulsant medication levels. Approximately 50% of the autopsies show neuropathological lesions; many autopsies showed no obvious cause of death [61]. Prolactin levels are not increased in these patients. Seizures could develop in subjects without known epilepsy secondary to drug toxicity, and their detection during sustained physical confrontations, especially if akinetic, might be quite difficult [54, 55].

## 4. Discussion

This literature review indicates that in-custody or in-prison deaths occur frequently. The causes for these deaths include natural causes secondary to underlying chronic diseases, homicide, suicide, accidents, and unexplained death. The causes of death depend in part on the type of inmate population under review. Inmates in prison tend to be older and die from natural causes; inmates in jail are younger, often have been in custody for only a few days, and frequently commit suicide [10]. One study reported a remarkably high suicide rate as an explanation for in-custody deaths. Regardless of the underlying cause, all deaths need investigation and explanation to the possible extent. These investigations are relatively straightforward when there is significant external and internal trauma and when there are underlying chronic diseases, such as coronary atherosclerosis resulting in acute myocardial infarction. However, some cases with death secondary to natural causes probably do not have underlying chronic disease and some deaths are unexplained or accidental. These latter cases require more extensive investigation. Histologic studies of myocardium and the identification of contraction bands and calmodulin can provide information about stress cardiomyopathy, and histologic studies of the lung with overinflation of alveolar spaces can provide information about asphyxia. Postmortem molecular studies can help identify stress cardiomyopathy (calmodulin), AMI (CCC9/SRT1), asphyxia (aggregates of surfactant protein A), and excited delirium (increased levels of heat shock protein 70). Both the presence and absence of these morphological and biochemical changes can guide the forensic evaluation of these deaths. Maeda and colleagues have reviewed the use of mRNA transcripts in molecular pathology and suggested that quantitative analysis of mRNA can support and reinforce morphological evidence [62].

Wangmo and colleagues have written two recent papers on the investigation of deaths in custody [1, 2]. They suggested that the situation of in-custody death automatically raises serious concerns about the effectiveness and fairness of any medical-legal examinations. They point out several important issues, including the lack of independence of investigators, conflicts of interest and biased perspectives, apathy towards the victims, and the quality of forensic reporting. They suggest that experts and authorities in this process need to establish a universal definition of death in custody. In addition, government organizations need to systematically collect quantitative data using standardized formats for these deaths. At present, the efforts to compile information on arrest-related deaths are incomplete [10]. In particular, the circumstances surrounding these deaths need to be detailed. The investigators need to be guaranteed independence, and this likely would require regional agencies to take over the investigations rather than local agencies. The expert task force on excited delirium recommended a national registry to record the numbers and circumstances surrounding these deaths [53]. In addition, they recommended development of a biorepository to collect organs from these decedents. Sims et al. have described an approach used for peer review and systematic evaluation of forensic pathology in the South Australia state forensic facility [63]. This process involves both informal discussion in the autopsy room and formal discussion after the autopsy has been completed. In addition, there is mandatory formal review of homicides, deaths in custody, suspicious cases, and pediatric cases. This process almost certainly improves the quality of the forensic investigation and the quality of report submitted by this office.

In summary, the investigation of in-custody and other related deaths, those such as secondary to torture, requires a comprehensive approach to identify the best explanation for the death. This approach should include a careful description of the circumstances surrounding the death, the behavior of the decedent using a checklist for characteristics related to excited delirium, a comprehensive medical history, drug and toxicology screens, and a forensic autopsy including both external and internal examination for trauma, underlying cardiac diseases, bleeding, and miscellaneous unexpected medical problems which could have contributed to the death. Semiquantitative histological studies can help identify victims who suffered mechanical asphyxia. Postmortem studies of cardiac tissue can identify calmodulin in decedents with stress cardiomyopathy, as well as specific patterns of CBN related to particular categories of cardiac injury. Immunochemical studies can identify increased amounts of surfactant protein A in decedents with asphyxia and heat shock protein 70 in decedents with excited delirium. These investigations will require additional time, cost, and expertise; these bodies should be transferred to regional centers for thorough study. Geographic centralization would allow for decreased bias, improved data reporting, and accumulative experience of regional forensic investigators.

## Figures and Tables

**Table 1:** Summary of in-custody death series.

Location	Time frame	*N*, % male	Natural death	Homicide	Suicide	Accident	Undetermined
Nebraska [4]	2003–2009	38; 79%	14	8	8	8, no details	0
Bexar County, TX [5]	1985–2010	133; 89%	78	36	9	8; 5, drug related (3 jail/2 arrest); 3, traffic accidents prior to arrest	2
Karachi [6]	2005–2010	61; 95%	36	13	7	5, in prison	0
Karachi [7]	2006-2007	14; 100%	12	2	0	0	0
England/Wales [8]	1978–1997	1631; 100%	723	30	840	33, no details but in prison	5
Ventura City, CA [9]	1992–1996	21; 100%	2	4	0	14, while in police custody	1

*Total*	**1985–2010**	**1898, 97.9%**	**865 (45.6%)**	**93 (4.9%)**	**864 (45.6%)**	**68 (3.6%)**	**8 (0.4%)**
